# Chronic Syphilis

**Published:** 1888-05

**Authors:** H. M. Lawson

**Affiliations:** Cuthbert, Ga.


### CHRONIC SYPHILIS.

BY H M. LAWSON, M.D., CUTHBERT, GA.

℞. Phytolacca dec. rad. p........................
Ferri hydrat. stramonii fol. p.................aaʒ ss,
Hydrarg. bichloride..........................gr j.

F. Pill No. LX. S. One pill after each meal and at bedtime.

The above is the be-t pill for chronic syphilis I ever used. Some may object to the Homœopathic quantity of the hydrarge bichloride, but I hope they will try the prescription before changing it. Professor Ricord says that in these cases he gives the iodide of potassium in normal doses so long as it tells on the disease, then increases it to a drachm or more a day; but in defiance to these large doses the treatment often hangs fire, the patient ceasing to improve, and that he then has recourse to some form of mercury with excellent effect.

If in the treatment of syphilis we interdict *all oleaginous articles of diet*, most, if not all, the hypermedication now often recommended may be avoided. The oleaginous element of diet is the one upon which the disease thrives, and if disallowed, fewer relapses and more prompt and positive results would follow proper treatment. If a case evinces inflammatory symptoms or eruptions on the skin, I add to the formula 6 or 8 grains of tart. antimony et. potass. the pill will prove sufficiently laxative if the patient is not of a constipated habit. The surgeon should see that the bowels are moved daily, and that too much purgation is not induced. I do not think the fluid extracts will answer so well; but if the roots and leaves gathered at the proper season are used, a successful result may be confidently expected.

